# Social resilience within the carescapes of Asian female migrant aged care workers

**DOI:** 10.3389/fpubh.2025.1565750

**Published:** 2025-04-14

**Authors:** Monika Winarnita, Carmela Leone, Thomas Klassen, Irene D. Blackberry

**Affiliations:** ^1^Asia Institute, Faculty of Arts, The University of Melbourne, Parkville, VIC, Australia; ^2^John Richards Centre for Rural Ageing Research, and Care Economy Research Institute, La Trobe University, Melbourne, VIC, Australia; ^3^School of Public Policy and Administration, York University, Toronto, ON, Canada

**Keywords:** aged care, female, migrant workforce, social resilience, carescape

## Abstract

Increasingly, Asian female migrants are playing a significant role in meeting Australia’s aged care workforce demand. This article analyses the lived experiences of Asian female aged care workers using the carescape concept, and a theory of agency to understand aged care access and workforce availability. It aims to identify the wider institutional and social structures that influence their agency and contribute to their social resilience as a critical member of the aged care workforce. Qualitative data were used for analysis; specifically, semi-structured interviews which were conducted with 10 Asian female migrant workers from the aged care sector. Analysis reveals that social and institutional structures both challenge and facilitate agency, and thus access to the aged care industry. The findings provide a deeper understanding of agency and highlights the social structures which contribute to developing social support networks and social resilience. Workplace policies and practices which facilitate the agency, adaptation and transformation of this workforce are important to understanding access to the industry and the retention of Asian female migrant aged care workers.

## Introduction

In 2020, 16 per cent of the Australian population were aged 65 and over. By 2066, older people are expected to make up 21–23 per cent of the total population (1). Due to Australia’s aging population, there is an increasing demand for aged care workers. However, Australia is experiencing chronic staff shortages in the aged care sector (2), with a shortage of at least 110,000 aged care workers expected within the next decade, unless urgent actions are taken to expand the aged care workforce (3). Migrants form a substantial and growing part of Australia’s frontline care workforce, and in 2020, 35 per cent of residential aged care workers in Australia were migrants from Culturally and Linguistically Diverse Background or CALD (4). Out of the 35 per cent of these residential aged care workers, data from the Australian Bureau of Statistics 2021 census shows that 80 per cent are from four identified Asian cultural background: Filipino 18%, Nepalese 25%, Indian 27%, and Chinese 10% (5). In 2022–2023, there was an increase in the number of migrant arrivals equating to an annual increase of 73 per cent, with most migrants arriving from India and China (6). In 2020, 86 per cent of the residential aged care workforce were females (4).

From 1976 to 2016, there was a significant growth in the proportion of care workers who were born in Southern Asia (7). While more recent figures are unavailable, the migration of workers from Southeast Asia has recently been encouraged by Australia’s Southeast Asia Economic Strategy to 2040, which includes recommendations for Australia to facilitate the movement of aged care workers, to meet workforce shortages (8). In addition, Australia’s Aged Care Industry Labour Agreement allows qualified direct care workers from overseas to work in the aged care sector, where appropriately qualified Australians cannot be attracted (9). Given the growing number of female aged care workers in Australia, it is important to understand the challenges they face in their recruitment and retention. This paper explores the lived experiences of 10 Asian female aged care workers from migration to employment in Australia, using the concepts of social resilience and carescapes. It adopts Giddens’ (10) Theory of Structuration to understand the structural facilitators and barriers which contribute to building social resilience.

## Literature review

### Migrant aged care workers

Migrant aged care workers in Australia face a number of ongoing challenges, including precarious work (11–14). This is due to contingent migration pathways, such as temporary skilled migration visa schemes (11, 15). Both migrant home care workers and residential workers experience underemployment, with exposure to casual employment increasing over time for those in the residential sector (11). Migrant aged care workers from non-English speaking backgrounds are more likely to experience underemployment and casual status than those from English speaking backgrounds (11).

Migrant aged care workers in Australia also face discrimination and racism (16–19). In a study of migrant care worker perceptions of Australian working conditions, workers from Africa and Asia were found to experience discrimination and racism due of the color of their skin and their accents (16). For female Asian aged care workers, gendered and racialized negative stereotyping of Asian women presents an additional challenge (14). This was highlighted during COVID-19, when Asians in Australia experienced increased discrimination (20, 21)—particularly in healthcare settings (22, 23)—with women receiving more racial abuse than their male counterparts (24).

Many migrant aged care workers are highly qualified, and despite workplace challenges, they mobilize existing skills and work experience toward building new careers (13). In a study exploring the lived experience of Asian migrant care workers in regional Australia, participants did not perceive distinct barriers to career development (14). Similarly, findings from an Australian study of migrant employment in the aged care sector highlights individual discourses about the meaningfulness and accessibility of work, even within in a wider context of gendered and racialized discourses which cast migrant women as being “well suited” to care work (13).

Research shows that racism and discrimination experienced by migrant aged care workers negatively impacted their health and wellbeing, however, effective leadership contributed to building inclusive and egalitarian environments, and peer and management support were identified as important in building cultural competence and inclusive environments (17). Supportive environments can contribute to retention and career progression. This was demonstrated in a study of migrants working in not-for-profit faith-based residential aged care, where the creation of safe environments and good working relationships contributed to a harmonious and egalitarian workplace (25).

### Carescapes of migrant aged care workers

The existing literature provides insights into the carescapes of Australia’s migrant aged care workforce. “Carescape” refers to the landscape or terrain of care (26) and considers the wider “relationship between policies, services and infrastructure related to care as determined by nation state, local government and employers” (27). Drawing on the carescape concept can direct analysis toward policies and practices relating to care, as well as care experiences, and the cultures of organizations and of care itself (28). Carescapes highlight the external structures, resources and services which influence care activities in time and space (29). As well as the practical delivery of care through services, carescapes include political and social conceptualizations of care, and recognize that care policies contain a historical and cultural dimension, and that practices of care are informed by social and political ideas around care (30).

Literature focusing on carescapes redefines the home as a landscape of care (31), and highlights under-resourcing and social isolation in the context of post-parental care for aging people with intellectual disabilities (32). Carescape literature has recognized the importance of place to residents of a Dutch aged care facility (33), identified spatial divisions in managing risk and safety in COVID-19 nursing care (34), and demonstrated the changing patterns of care over time for grandfathers (35). In the literature, agency is considered in relation to aspects of carescapes, such as the employment pathways of migrant aged care workers in Australia (13), the landscapes of care for older adults with Sri-Lankan-Australian trans-national families (36), and healing landscapes of aged-care gardens (37, 38). However, the interrelations of agency and structure and their contributions to social resilience within the carescapes of Asian female migrant aged care workers in Australia have not been explored.

### Social resilience and migrants

Social resilience refers to the social factors needed for individuals to cope, adapt and transform, especially during a crisis (39). Resilience is intrinsically related to social practices (40, 41) and social experiences (42). For migrants, social resilience is an ongoing process of adjustment determined by assets and resources—all of which take time for migrants to access (43). Social resilience includes the continuous development of skills and the acquisition of knowledge and attitudes which accumulate over time (44). In the case of refugees, social resilience plays a critical role in their adaptation and resettlement process, and can include feelings of being welcome, having access to social services, establishing new relationships, and opportunities for education (45). As a concept, social resilience is an important mechanism for understanding how adaptation and coping can contribute to stability, even in situations of social change (46). A social resilience approach recognizes that institutions play a critical role in the lives of migrants and their efforts to establish their lives (47). It emphasizes the relationship between individuals and institutions and highlights the role of agency (47).

### Theoretical framework

This study adopts Giddens’ (10) Theory of Structuration to interrogate the interrelations of agency and structure and how they contribute to the social resilience of Asian female migrant aged care workers. It considers the roles of agency and structure with respect to the ability of Asian female aged care workers to cope, adapt and transform as they navigate their journeys from migration to employment. The use of agency as a lens is familiar in Australian migration studies (13, 48–50). However, in focusing on agency and structure in the context of the carescape, this study establishes a new understanding and provides a more balanced analysis of agency. In the carescapes of study participants, institutional and social structures include aged care policies, and political and cultural ideas around aged care, as well as socio-cultural norms and expectations. Consideration is given to the prevailing practices, care experiences, workplace cultures, and the resources and services available, particularly with respect to workforce retention and professional development.

According to Giddens (51), “agency refers to doing” and to the capability of a subject to produce an effect. Agency is linked to structural influences, which can include material resources, the expectations of others, and internalized views relating to behaviors (52), as well as external structures, which can restrict agency (53). For an individual to have agency, Giddens considered that he/she “must act intentionally… have the capacity to act on his or her intentions… and have the power to create a new event or to intervene in an existing event” (54). This paper thereby explores the intentionality, capacity, and power of Asian female migrant aged care workers to act in ways that develop social resilience. It contributes to the aged care debate by answering the research question: ‘How do Asian female migrant aged care workers in Australia exercise agency toward building social resilience within their carescapes?’

## Methods

Analysis was conducted using data from a mixed-methods study which explored the recruitment and retention of migrant female aged care workers in rural, regional, and urban Victoria (the State of Victoria in Australia), specifically focusing on how social resilience was built through support from the workplace. Victoria was chosen as it is one of the most culturally and linguistically diverse states in Australia. In addition, Victoria’s share of older Australians is the same as its share of the total Australian population, with suburbs in outer Melbourne having the largest number (3). According to the Australian Bureau of Statistics (6), in urban areas of Victoria (particularly the city of Melbourne), Mandarin has experienced the highest growth in speakers, with Cantonese, Punjabi, Hindi and Filipino/Tagalog being the other Asian languages showing increases. This is reflected in the list of our participants’ country of migration (see Table 1).

**Table 1:** Participant characteristics.

Name (de-identified)	Country of migration	Age	Years working in aged care
Meili	China	Over 40	Over 10 years
Lin	China	–	Since 2014
Jaya	Nepal	–	–
Divya	Nepal	–	Since 2011
Rosaria	Philippines	50	Over 13 years
June	Philippines	35	Since 2012
Chen	China	35	Since 2008
Sheri	China	37	Less than a year
Udaya	Sri Lanka	–	2009–2011
Sati	India	32	–

This study received approval from the (La Trobe University) Human Research Ethics Committee (Ethics number is HEC18378) in 2018. The study’s online survey content outline and the interview question scripts were not tested in any pilot but they were provided to the university’s Human Research Ethics Committee for approval with all amendments made as per the Ethics Committee’s reviews and suggestions. A final draft was provided to the Committee prior to a HREC approval number being given and displayed in recruitment information and flyers.

The online survey comprised of 30 questions on Qualtrics that recruited 117 participants through contacts that the researchers have in the aged care industry. Flyers sent by email to a publicly available list of aged care provider contacts in the state of Victoria, aged care industry associations and the Ethnic Community Council of Victoria. Information documents and flyers about the study contained a link to the online survey that the participants could then access. Recruitment of the participants was therefor intentional, targeting workers in the aged care industry through aged care industry contacts. The recruitment information specifically asked for an inclusion criteria of persons identifying as female, from a migrant or Culturally and Linguistically Diverse (CALD) background and working in aged care. Therefore, the study excluded any participants who did not have those three criteria (female, migrant/CALD, aged care workers).

A follow-up semi structured phone or face to face interview of 20 participants who consented to be interviewed (as a direct question) through the online survey were then conducted. There were no criteria to delimit the number of interviews as there were only 20 out of the 117 participants who participated in the online survey willing to provide their contact details on the form. These participants consented to have a follow up interview on Qualtrics. The majority of the interviews were conducted by phone as this was the contact detail they provided on the online survey. One participant agreed by phone for the researcher to interview them in a home visit setting. Four participants based in Melbourne agreed to have a face-to-face interview at the university (a neutral setting), which was close to where the participants live and work as per their initial phone contact with the researcher. Ten of the participants were Asian migrants; their interviews provide the qualitative data for this article that focuses on the Asian female migrant aged care workers experience. The five participants that had a face-to-face interview (at home or the university) have an Asian background. The characteristics of the participants are detailed in Table 1.

**Figure 1:**
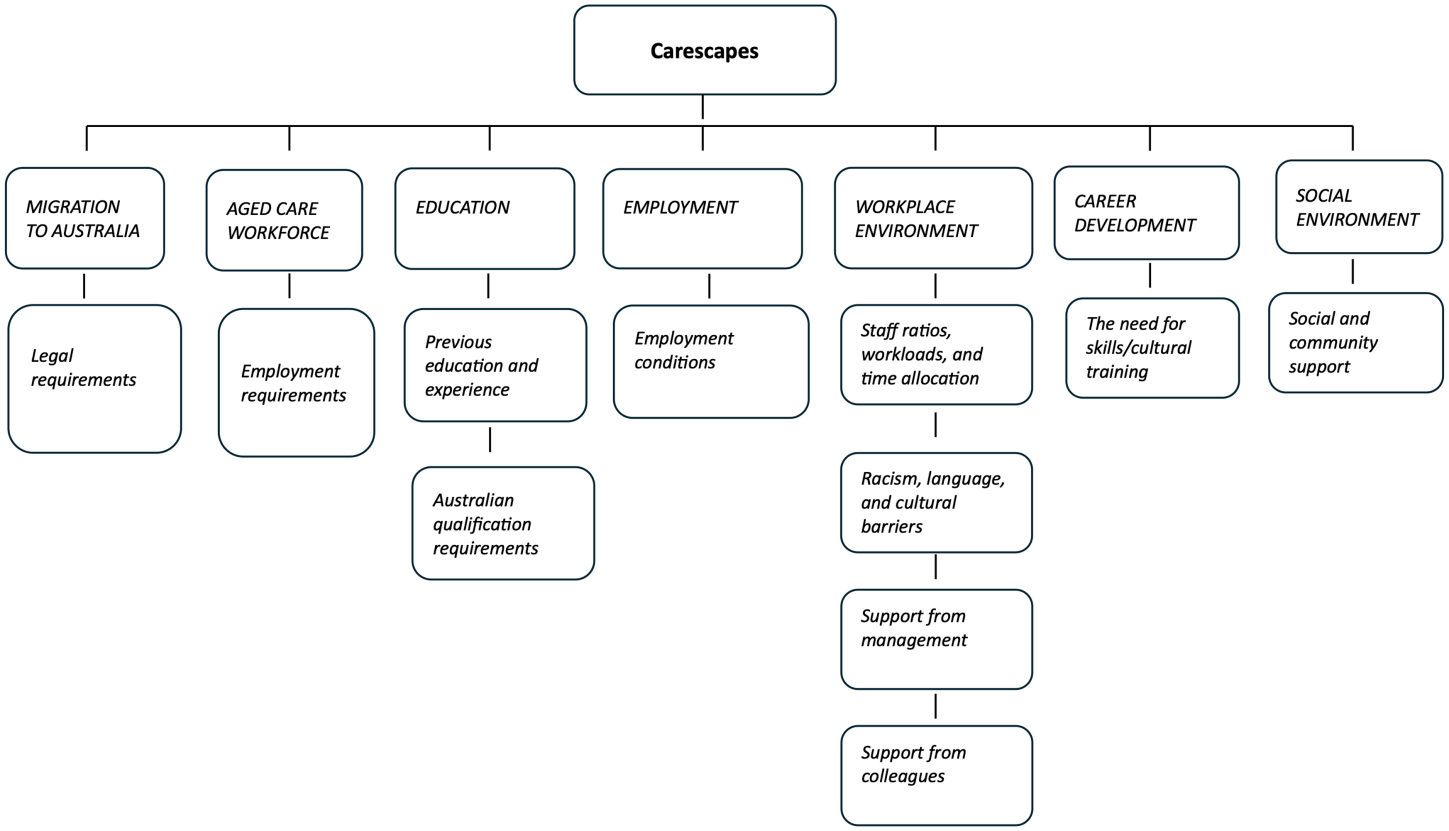
Coding tree.

The 10 semi-structured interviews were transcribed (see Supplementary Appendix 1), and interview data were coded in NVivo. The objective of the data analysis was to identify how Asian female migrant aged care workers in Australia exercise agency toward building social resilience within their carescapes. Data were organized in the first instance using the carescape concept, to map the terrain in and through which participants navigated their journeys into the age care workforce and within. This involved identifying the various structures and environments, from the participant’s journey to Australia, their entry into aged care and their employment. Data was then analyzed inductively, using Giddens’ (10) Theory of Structuration to identify structural barriers and facilitators to agency within the carescapes. Analysis thereby focused on the actions of participants. In the analysis, intentionality refers to the intentional actions of a subject, being “an act which its perpetrator knows, or believes, will have a particular quality or outcome and where such knowledge is utilized by the author of the act to achieve this quality or outcome” (51). Capacity refers to the ability of a subject to achieve their desired outcomes (55). Lastly, power refers to the capability of a subject to make a difference, including the ability to deploy causal power, and to influence powers deployed by others (51). The participants’ carescapes constitute the top level of the coding tree, with the identified themes relating to the structures and environments in which participants exercised agency, beneath (Figure 1).

Lastly, data were interpreted drawing on the three capacities of social resilience—coping, adaptive, and transformational capacities, as defined by Keck and Sakdapolrak (39)—to identify how social resilience was built through support within the various structures and environments of their carescapes.

## Findings

### Migration to Australia

#### Legal requirements

In all carescapes, participants migrated to Australia with intentionality. While circumstances may have directed participants toward migration, when they acted, they did so with the intention of producing certain effects, such as to establish a life with their husband/family, to improve their life circumstances and/or to pursue their careers. In their migration journeys, the capacity and power of participants to act related to meeting the conditions of visa programs. Because participants had to fulfill application requirements, their carescapes were influenced by the institutional structure of Australian migration law. Most participants migrated to Australia on partner visas, and their power may be perceived as challenged by their reliance on their husband’s citizenship status. However, one participant (Jaya) sponsored her husband’s migration to Australia, and two participants (Chen and June) migrated on international student visas.

### Aged care workforce

#### Employment requirements

All participants had intentionality in entering the Australian aged care workforce, though the ways in which they gained entry varied, particularly with respect to their capacity and power to act. Resources which facilitated the agency of participants to pursue their careers in aged care related to their previous education and employment, or to gaining Australian aged care or nursing qualifications. For some participants, agency was also facilitated via an introduction to the idea of working in the aged care sector, as well as support and encouragement from others, as relational agency. Relational agency involves “people producing particular effects in the world and on each other through their relational connections and joint actions” (56).

### Education

#### Previous education and experience

Some participants were working as nurses or studying nursing before migrating to Australia. Their previous experience and education were resources which facilitated their progression into the aged care sector and provided them with the capacity and power to move toward achieving their career goals. All participants were required to complete Australian qualifications to work in aged care, and some participants recognized the opportunity for new learning to facilitate their agency toward achieving career goals. June who was working as a nurse in the Philippines, recognized that working in aged care in Australia would develop her skills, as she was undertaking more responsibilities than in her previous role:

‘The nurses [in the Philippines] are expected to give medications… follow-up, liaise with doctors’ orders and all that. Here it’s actually everything… from personal hygiene, showers, medications, wounds, everything. So that alone attracted me already because I said, oh it’s learning, it’s skills. You get to upgrade’ (June).

June saw that learning new skills would provide her with the capacity to facilitate upward mobility (at the time of the interview, June’s role was clinical manager, temporarily acting as residential manager). Similarly, Sati, who held a nursing degree in India, recognized the opportunity for learning new skills to progress her career. She undertook a Master of Clinical Education rather than entering nursing directly: “I felt like I needed more experience, I needed to learn what happens, everything in the Australian culture, what happened in the Australian hospital industry. So, I chose to go back to scratch and start doing my studies again” (Sati). Undertaking a higher qualification facilitated Sati’s capacity to pursue her career goals: “I’m doing my master’s in clinical education, because I love to train people” (Sati).

Udaya, who worked as a nurse in Sri Lanka, also saw learning new skills as a way of progressing her nursing career. She studied to work as a Personal Care Assistant (PCA), before becoming a Registered Nurse (RN): “After my PCA course they offered me a job as a PCA there. Then by the time when I finished my nursing course, they offered me as an RN” (Udaya). Udaya was pregnant while working as a PCA, and nursing facilitated her agency to obtain more suitable work:

‘When I got my registration, they offered me a casual job because I think I was pregnant that time, so I don’t want, I didn’t want to go full-time… It was physically and yeah, it was hard… Because it was a high care facility. So, everyone, almost everyone needs machine, lifting machine, standing, you got the thing. So that’s why I thought I might just work a little bit then start RN straight away’ (Udaya).

Australian qualifications also facilitated the agency of Rosaria—who was a nursing student in the Philippines—to work in the aged care sector when she was unable to continue studying nursing in Australia. When Rosaria was looking for work, her friends working in aged care supported her application for a job in the sector. This support enabled her to cope and adapt to her circumstances. Rosaria’s nursing knowledge helped her obtain her Certificate IV in Aged Care, and she then gained employment through her work placement. For Rosaria, social support facilitated her agency, and her qualifications and work placement provided her with the capacity and power to move toward achieving her career goal of working in the healthcare sector.

#### Australian qualification requirements

Meili had no previous experience/education in nursing. Obtaining qualifications in Australia provided her with the capacity and power to achiever her goal of becoming a nurse and enabled her to adapt to her new role. Meili held a Bachelor of Economics and had worked as a faculty administrator in a Chinese university before migrating to Australia. Meili had desired to become a nurse, however, she had studied Economics in China because of her parents: “Back home… when you go to higher education, majority of the time, your parents decide what you are going to do” (Meili). Social structures in China had impeded the achievement of her goals. However, while studying English in Australia, Meili was supported by her teacher to work in aged care, as a pathway to nursing. This support, together with her Australian qualifications, facilitated her agency to become a nurse and enabled her to cope and adapt to achieving her goals.

For another participant, Sheri, her entry into aged care was directed by a separation agreement with her husband. Australia’s marriage laws, as institutional structures, were a factor in potentially challenging her agency. Nevertheless, her actions were intentional as she entered aged care for her own reasons and developed her skills toward achieving her career goals. Sheri had completed a Bachelor of English Communications in China and had worked as an editor and a receptionist. However, her husband suggested three jobs: “Aged care, health care and hairdresser… apparently these three jobs are easier to get, and also, they are in high demand…I decided to do aged care, because I like older people” (Sheri). Sheri’s entry into aged care was as a PCA and she was undertaking a Diploma of Health to advance her career: “I like aged care, that’s why I want to stay in the industry, but I want to work in a different role… I want more opportunity to communicate with people because that’s the most enjoyable part to me. So, I’m doing this course. I’m hoping to change my job after a year when I finish the course” (Sheri). Sheri’s completion of a Certificate IV in Aged Care and her subsequent undertaking of a diploma provided her with the capacity and power to advance professionally in the aged care sector. Her ability to cope and adapt to the conditions of her marriage separation allowed her to transform and achieve her goals.

### Employment

#### Employment conditions

While all participants had intentionality in seeking employment, casual work either facilitated or challenged the capacity and power of some participants to pursue their careers and a life-work balance. Not all participants had the intentionality to work on a casual basis. When Meili began working in aged care, she was a casual worker, however, when the opportunity for a part-time role arose, she applied for and obtained the position. Part-time employment provided Meili with the capacity to become a nurse while achieving a work-life balance—giving her the ability to adapt to her circumstances. For some participants, however, casual work allowed them to manage their lives and achieve their career goals—thereby providing opportunities to move beyond coping to adapting to their circumstances. Rosaria chose to work part-time. Sheri worked on a casual basis because it suited her and her circumstances with two children. Similarly, casual work suited Udaya while she was pregnant. In these carescapes, economic influences and aged care sector policy and practice regarding employment acted as institutional structures.

### Workplace environment

#### Staff ratios, workloads, and time allocation

Within carescapes, all participants acted with intentionality, however, institutional structures sometimes impeded their capacity and power to act. Udaya identified difficulties associated with the amount of time allocated to tasks, as well as workload issues: “Within that eight hours we have to do almost everything… So, it’s like too [much] stuff to do within that period. It’s quite challenging. Sometimes yeah, we are not taking our breaks. We just want to work, work, work then finish it” (Udaya). Similarly for Sheri, the time allocated to complete her tasks challenged her agency: “The most rewarding part of this job is when you talk to them and make them happy. That’s why I like this job” (Sheri). For these participants, institutional structures which assert staff ratios, workloads, and time allocation for jobs challenged their agency to work in ways that were rewarding, and which attend to their wellbeing, and their ability to adapt and transform.

#### Racism, language, and cultural barriers

For some participants, racism, and discrimination challenged their capacity and power to act, and social structures—founded on historical and cultural perspectives—were influential in some carescapes. Meili identified racism from residents and their families as challenging. During COVID-19, Lin experienced racism in her workplace, where she was treated differently from colleagues who were not of Asian descent. Lin also experienced increased aggression from residents during that time. Udaya had difficulties with Anglo-Australian colleagues in the beginning of her employment and also identified families of clients as challenging: “Even though we did a super good job, sometimes they… I feel like some families very racist. Like they just see you colour… I feel like some families are very, very challenging*”* (Udaya). Udaya viewed support from her management as potentially facilitating relational agency in these situations, should she need help: “We can always go and talk to my manager because she’s really good… if you have racism or if you feel like you got like discrimination or something like that… I straight away go to managers if I do have a problem like that… Yeah, so she will come straight away” (Udaya). Relational agency contributed to the establishment of support networks for Udaya, and to building social resilience.

Diverse cultures and workplace social structures were identified as challenging participants’ agency as they impeded communication and the completion of tasks. However, Meili felt she understood her Anglo-Australian co-workers because of her Australian husband, and she viewed her colleagues as supportive. Nonetheless, she recognized challenges faced by workers from migrant groups due to cultural differences, and the need for adaptation both in terms of actions and the broader carescape:

‘If you’re multicultural, how do you adapt to Australians? If you’re Australian, how do you adapt to mainly about, from multicultural background, mainly talking about how multicultural people adapt to the other multiculture, or your environment… How do they think? How are they going to do, if things come, what are their strategies? What do they think of it?… If it’s a language issue, it just comes to how do you communicate… If you don’t communicate, you don’t know what they’re thinking, what they do, what they’re thinking, is there a problem or anything’ (Meili).

Meili identified the need for managing multicultural differences in the workplace with respect to action and problem-solving, to facilitate understanding and cohesion, and relational agency.

While participants had intentionality in their actions, their capacity and power to act was sometimes challenged by language and communication barriers, which acted as structural forces. As Jaya identified:

‘It’s very hard to communicate with the person who is not from your one country, who is from the different part of the country. It’s very hard because we don’t have the same language and communication… the worker from the other country, the other place, they are trying to say something, and we just understand something. Whenever we try to say something, they just understand other things. There is this communication problem. And sometime[s]we just have some problem with the resident as well because we just try to do something, we just try to help something, but they just take it in a wrong way’ (Jaya).

For Chen, getting to know the client helped with communication issues: “We say something, and they think of another thing. When we get to know each other and then we do not make any mess of each other. We know we might make a mistake so we can, we make clear for each other” (Chen). Through finding new ways to communicate with clients, Chen was able to adapt, and even transform her relationships.

June identified communication breakdowns between residents and workers as impeding good care, and therefore to her capacity to act. Good communication was seen to facilitate agency, as June worked to problem-solve in these situations, and to thereby adapt to the situation:

‘If I know that there is communication breakdown for whatever reason and they care of the resident is compromised, I would always call them in. Would always call my staff in to say, what are we doing, why is this happening, how can we resolve this, how can? … and it’s about conflict resolution and all that. So, promoting communication. I have a staff who are struggling with the English, like they have to take English class. And whenever I can or whenever there’s a few people who know how to help out with the English then we try to help’ (June).

For Sati, knowing a client’s culture was important when dealing with resistance: “I’ve found that I’ve learned different things about different cultures, what they would prefer when they are palliative” (Sati). Sati considered experienced staff members as an asset in problem-solving: “They work pretty quickly on anything that could lead to any serious miscommunication or serious incident” (Sati). Understanding different cultures facilitated Sati’s capacity to work in a multicultural environment, and to provide good care to multicultural residents. Cultural understanding also contributed to building social resilience, as Sati was able to adapt according to the needs of the clients.

#### Support from management

Several factors facilitated the ability of participants to act in their carescapes. This included support from management, which was viewed as important by several participants and demonstrated in different ways. In the carescape of Sati, institutional structures—as organizational responses—supported her agency toward working in an inclusive environment, as language and cultural barriers were addressed through the role of a people and culture officer:

‘We do have specifically people and culture officer, he organises different sessions, information sessions and seminars as well to promote a multicultural environment. They give counselling and everything, if you feel in any way that you are not comfortable working with anyone, or not comfortable working in that environment’ (Sati).

Support and leadership from management were identified as also providing participants with the capacity and power to act in their workplace. This was demonstrated in Sheri’s carescape, as she was supported by her manager when she wanted to advance her career and work in the lifestyle area. Her observation of the role was that it was “more like a white people role” (Sheri). Sheri’s manager facilitated relational agency by encouraging her to challenge the social structures which could have potentially impeded her capacity to become a lifestyle worker: “I actually talked about it with my manager… He said if you want to do it, just do it… He’s really encouraging, that’s why I think he’s a great leader” (Sheri). Support from management empowered Sheri to achieve her career goals. Similarly, support from Chen’s manager empowered her and facilitated her agency toward improving her pay:

‘Because my manager help me to increase my salary. They provided an opportunity for me, like study a Certificate IV [in] Home Care and Disability. And they make a requirement for the city of Yarra council, they say I have this certificate and then I have worked there so many years, and they helped me to increase my salary’ (Chen).

By providing Chen with the opportunity to acquire a qualification and advocating for her with the local council, her manager facilitated capacity and empowered her to achieve her career goals. In both these instances, support from management contributed to building social resilience, as both Sheri and Chen found ways to cope and adapt to the challenges that they faced in progressing their careers.

June was also empowered by management when she first started working in aged care: “My manager at that time… they were really lovely. They were more of empowering their staff as well. Not only the senior ones, but also the new ones who came… I did not feel ostracised… I did not feel like I was different. I was always considered as one, as part of the team” (June). June also experienced empowerment through guidance and mentorship from her colleagues:

‘There were actually a lot of Anglo-Australians… they were actually the ones who taught me how to do things… they were the ones who actually said, just because you’re new, just because you’re from Asia and you think this, this, this, people can just trample all over you or can bully you, don’t let them bully you, don’t let them this, this, this. You’ll learn your job and you learn it pretty well so that they will not have the chance to bully you, or they won’t have the chance to criticise you because you’re good, you’re doing your job well. That’s how they taught me, that’s how they mentored me’ (June).

Support from June’s manager and her colleagues meant that she was able to cope and adapt to the circumstances of her workplace, and even to transform, through being empowered.

#### Support from colleagues

For some participants, learning and support from colleagues facilitated a relational agency which empowered them to carry out tasks, and build social resilience. In June’s carescape, social structures enabled her capacity and power to act, as a workplace culture of inclusion facilitated her individual agency when she was starting her career in aged care:

‘The job becomes difficult if you don’t form relationships with your teammates or with the residents. But I was able to do it. I was able to work well with my team leaders, the clients, and the people who I worked with were just really lovely, too… I didn’t really feel like I was any different because I wasn’t made to feel different’ (June).

With support from her colleagues, June was also able to cope and adapt. For Sheri, cultural understandings between fellow Asian migrant workers helped her navigate communication issues: “Because we are mostly from Asia… So, we all sort of understand, because we are not from Australia, there are things we experience. So, we understand not to say something, or to say something” (Sheri). Sheri consequently felt supported by colleagues, which facilitated relational agency and helped her to cope and adapt. Sheri felt that if she encountered a difficult situation she could not manage on her own, she could ask a colleague to help her: “I have lots of support from them. So that’s why I like working here too” (Sheri).

### Career development

#### The need for skills/cultural training

Skills training was identified as providing participants with the capacity and power to advance their careers, and several participants expressed a desire for more skills training. Meili recognized that her work placement provided her with skills that gave her confidence to carry out her job in aged care. June saw nursing in Australia as an opportunity to advance—to “upgrade,” through learning new skills, and credited good training for doing her job performance: “The Filipinos have been there for a long time and those who have, those who have the senior staff there helped me to learn and do my job well” (June). In all carescapes, participants worked with colleagues and residents from multicultural backgrounds, and many expressed a desire to undertake cultural training, toward building social cohesion, improving workplace relations, and ensuring the completion of tasks. Participants recognized that cultural training would facilitate capacity and power in their everyday work environments. Both Lin and Jaya expressed a desire for language and cultural training/training to gain a better understanding of diversity in their workplaces. Divya and Meili acknowledged barriers associated with diverse cultural values. Divya saw cultural differences as an obstacle to the completion of her tasks: “We have to work together. And if there’s just some different way to work, or different way to think, it will clash. So, it will be hard to finish off, complete the task. So, that’s the main challenge” (Divya). Training was recognized by participants as a resource for facilitating agency and for helping them to cope and adapt in the workplace and potentially transform.

### Social environment

#### Social and community support

Outside of work, support systems for participants were diverse and included friends, family, and geographical, as well as cultural communities. These support systems facilitated social and cultural integration and community connection for participants, and acted as social structures which helped them to cope, adapt and transform, and contribute to capacity and power in their carescapes. The support systems of participants were diverse. Meili and Lin were friends outside of work, and they provided support for one another, including childcare. Udaya made friends since coming to Australia and has had support from her husband, his friends and family. Rosaria’s support network was her Filipino community. Sati was supported in her neighborhood by multicultural events held by her local council, and programs in libraries which included language education and support.

June saw support systems as being especially important to the adjustment of new migrant aged care workers to a different culture: “If you have a good, strong support system around you I think that coming into a new country and adjusting to a new culture would not be as challenging and difficult as everyone thinks. I had a very good support system, both outside and inside work… that’s why for me it wasn’t that difficult” (June). For Sheri, establishing social support systems outside of work was challenging, following her separation from her husband. However, her employment in aged care facilitated her capacity to build a life for herself: “At the beginning of my separation, it was pretty hard, and I got help from my kids’ friends’ family a lot. And after I got the job it’s much easier because I can decide when I work, and I have a special time” (Sheri). While Sheri was supported by Asian colleagues at work, she had no social connections with the Chinese community because of her marriage status:

‘My family is not here, and they only knew about my separation after I settled everything down. I don’t have many friends here. I just got separated and in China a separated woman, they see you very differently. So, I don’t want to experience that. So, I’d rather not go to a Chinese community and be judged’ (Sheri).

Chinese culture—as a social structure—impacted her social situation in Australia and her agency within her community.

## Discussion

Through the exploration of agency and the role of structural influences in Asian female migrant aged care workers’ carescapes, this study contributes new knowledge about how agency is exercised in relationships and within social structures. The findings complement existing research which highlights workplace challenges such as racism, discrimination and precarity of work. This study also demonstrates that structural forces do not always challenge agency, as where institutional or social structures aligned with the aims of the participants, they facilitated agency. This challenges common notions that structures act in opposition to human agency (57) and contributes to the literature an argument for the false dichotomy of the individual versus society. The mandatory requirements of Australian immigration law and aged care employment could be considered to impact the voluntarism of the participants, and therefore their agency. However, even under the authority of both institutional structures, the participants acted with intentionality to achieve their desired outcomes. Institutional structures facilitated their capacity and power, as they had the ability to achieve their goals of migration and employment, and the capability to make a difference in their lives and careers in Australia.

Similarly, casual work—which is commonly associated with exploitation and precarity for migrant aged care workers and thereby as challenging their agency (58)—aligned with the life and career goals of some participants. Certainly, not all participants wanted to work on a casual basis, but for those who needed flexibility of employment—such as Udaya who was pregnant—it provided them with the ability to achieve their desired goal of balancing their work and home life, and the capability to affect change in their lives. The structural forces which determine casual employment challenged and facilitated the agency of participants, depending on their needs, toward achieving their career goals and managing their life-work circumstances.

Where the desires of participants and the requirements of institutional structures did not align, however, their capacity and power were challenged. Institutional structures which have authority over staff ratios, workloads, and time allocation for jobs challenged the agency of some participants to achieve their goal of rewarding work. In that these factors impacted the ability of workers to take their breaks—which are an entitlement under the Aged Care Award 2010 (59)—institutional structures also challenged their rights, highlighting tensions between institutional structures which assert working conditions (workplace practices) and those which grant entitlements to employees (awards). Importantly, neither of these supportive institutional structures facilitated the capacity and/or power of participants to act in their carescapes.

Where participants received support from friends, family, co-workers or management, there was a relational agency, which played an important role in facilitating action toward building social resilience. In these instances, relational connections, and sometimes shared actions, created capacity and power for participants, and allowed them to adapt and even transform. This finding further challenges common dualistic notions of agency and structure as within instances of relational agency, external structures are not confronted to enact agency, but rather, social relationships constitute “the very structure and form of agency itself” (56). Furthermore, the findings contribute to theories of agency, as while relational agency is commonly viewed as sitting in opposition to Giddens’ conceptualization of individual agency—which is considered to be primarily about reflexivity and not relational connections (56)—both individual and relational agency were observed in the carescapes of participants. This was best demonstrated in June’s carescape where her (individual) agency was facilitated by support from management and her colleagues (relational agency). In addition, June’s agency was facilitated within a workplace culture of inclusion and empowerment (social structure). June’s agency was thereby facilitated by the interactions of relational agency, individual agency, and social structures, and she was able to not only cope and adapt, but to transform from her general nursing role in the Philippines to a management position at an aged care facility.

Social structures both facilitated and challenged agency in participants’ carescapes. For Meili, social structures—as the cultural values of a community—created tensions between her career goals and the wishes of her parents in China. However, Meili’s migration to Australia facilitated her agency to pursue her nursing career goals. In the carescape of Sheri, social structures of identity and cultural inclusion provided her with the capacity and power to act in her workplace, as the support of her Asian colleagues enabled her to enact a relational agency, and to cope and adapt. Relational agency and social structures which facilitated workplace and community support contributed to Sheri’s social resilience. Outside of work, however, Sheri had no social connections with Asian people because of her separation from her husband. Social structures, in the form of Chinese social values and cultural exclusion associated with divorce stigma, challenged Sheri’s agency in her social life.

Social structures in the form of racism and discrimination also challenged the capacity and power of some participants, as historically and structurally embedded attitudes and assumptions of residents and colleagues impacted their ability to carry out their work. As reflected in research which shows that effective leadership from management can contribute to inclusive workplace environments (17, 25), support from management facilitated a relational agency for Udaya, which helped her to build social resilience. In Udaya’s carescape, the opposition between institutional structures as the authority and legitimacy of the manager, and the social structures of racism and discrimination were highlighted—as agency was facilitated by institutional structures while challenged by social structures. Similarly, where language and cultural barriers (social structures) challenged the agency of participants to perform care work, institutional structures asserted authority through the role of a people and cultural officer’s and the provision of education, information, and counseling, to facilitate agency. This finding further contributes to agency theory in challenging notions that all structures are uniformly opposed to agency.

While institutional structures such as migration law and workforce requirements/qualifications are unlikely to change, those which assert staff ratios, workloads, and time allocation for jobs—all of which are common issues cited in the aged care literature (60)—can be improved. The improvement of workplace policies and practices is important, to facilitate the agency of workers as well as their ability to adapt and transform within the workplace. Furthermore, social structures which facilitate the agency of participants, and which contribute to their social resilience can be fostered. This includes support from management which has been demonstrated to tackle racism and discrimination. Importantly, the needs of participants—as skills training and cultural education—provide a focus for improving workplace experiences and for the retention of migrant aged care workers.

With respect to guidelines addressing sex and gender in research (61), this study was not designed to analyze sex and/or gender differences (62). Given that 86% of aged care workers in Australia are female, only females were interviewed to highlight their experiences, but no sex and gender analyses were conducted as part of this study (see Supplementary Appendix 1). However, the authors recognize that aged care work is a feminized form of care labor in Australia (63), particularly for Asian female aged care workers (64). Further study will be needed to address this limitation of the research design and to provide a thorough analysis addressing the sex and/or gender differences in a feminized form of care labor where the majority of workers are female.

## Conclusion

That social and institutional structures both challenge and facilitate agency contributes to current theories of agency in challenging the false dichotomy of agency versus structure. In participants’ carescapes, social and institutional structures did not uniformly act in opposition to agency, and sometimes they opposed one another to facilitate agency. The findings contribute to a more comprehensive understanding about the agency of Asian female aged care workers in navigating from migration to employment. They highlight the significance of relational agency and social structures in building social resilience—through the development of community and workplace support networks—that supports their career development and retention in the aged care workforce. This study’s strength is the participation of Asian female aged care workers, and their contribution to research about care worker experiences in Australia’s aged care sector. The experiences of Asian female aged care workers, however, may not reflect or align with those of migrant aged care workers from other backgrounds. More research is required to determine the transferability of findings. Workplace policies and practices which facilitate the agency, adaptation and transformation of workers are important. This study suggests that support from management, skills training and cultural education can improve the workplace experiences and contribute to the retention of Asian female migrant aged care workers in Australia.

## Data Availability

The original contributions presented in the study are included in the article/Supplementary material, further inquiries can be directed to the corresponding author.
