# Supplementary material for: Social resilience within the carescapes of Asian female migrant aged care workers
Source: Front Public Health. 2025 Apr 14;13:1565750. doi: 10.3389/fpubh.2025.1565750 (PMC12045102; doi:10.3389/fpubh.2025.1565750)
Supplement: Supplementary file 1 [file Data_Sheet_1.pdf]

RECRUITMENT AND RETENTION OF MULTICULTURAL FEMALE CAREGIVERS IN AUSTRALIA'S AGED CARE WORKFORCE INDUSTRY: EXPLORATION OF SOCIAL RESILIENCE

Interview schedule

The research staff to introduce herself. Explain the aim of the study and obtain consent for interview and recording.

1. Tell me about yourself (e.g. age, background, culture, country of birth, languages spoken, education attainment, training in aged care in Australia).

2. What brought you (to work) here (rural/regional/urban area)? Please share your (migration) journey (e.g. how long have you lived in Australia, what work/education/family context).

3. Please describe your current and past role/s within the aged care industry including type of employment, employment level, hours of work, visa restriction, length of employment.

4. Can you describe your workplace environment e.g. community vs residential aged care; public vs private; culture specific vs mainstream? (recruitment, how culturally diverse is the workplace, working relations with your colleagues, other aged care workers/caregivers, managers and general staff).

5. What are some of the challenges you have encountered in providing care for the elderly? (e.g. communication, guidelines/standard, lack of leadership, further training).

6. How do you solve issues and problems you encountered in the workplace? How does your workplace help you solve issues or any problems you have so far encountered?

7. What types of support services are available for you as a multicultural caregiver and how do you access them? (e.g. CALD community groups, religious organisations, multicultural organisations)

8. From your experience, what are some of the key factors to recruit and keep multicultural female caregivers in the Australian aged care industry?

Thank You
